# Spatiotemporal slip distributions associated with the 2018–2019 Bungo Channel long-term slow slip event inverted from GNSS data

**DOI:** 10.1038/s41598-021-03982-6

**Published:** 2022-01-10

**Authors:** Yukinari Seshimo, Shoichi Yoshioka

**Affiliations:** 1 Department of Planetology, Graduate School of Science, Kobe University, Rokkodai-cho 1-1, Nada Ward, Kobe, 657-8501 Japan; 2 Present Address: Research Center for Urban Safety and Security, Kobe University, Rokkodai-cho 1-1, Nada Ward, Kobe, 657-8501 Japan

**Keywords:** Seismology, Tectonics

## Abstract

Long-term slow slip events (L-SSEs) have repeatedly occurred beneath the Bungo Channel in southwestern Japan with durations of several months to a couple of years, with a recurrence interval of approximately 6 years. We estimated the spatiotemporal slip distributions of the 2018–2019 Bungo Channel L-SSE by inverting processed GNSS time series data. This event was divided into two subevents, with the first on the southwest side of the Bungo Channel from 2018.3 to 2018.7 and the second beneath the Bungo Channel from 2018.8 to 2019.4. Tectonic tremors became active on the downdip side of the L-SSE occurrence region when large slow slips took place beneath the Bungo Channel. Compared with the previous Bungo Channel L-SSEs, this spatiotemporal slip pattern and amount were similar to those of the 2002–2004 L-SSE. However, the slip expanded in the northeast and southwest directions in the latter half of the second subevent. The maximum amount of slip, the maximum slip velocity, the total released seismic moment, and the moment magnitude of the 2018–2019 L-SSE were estimated to be 28 cm, 54 cm/year, $4.4 \times 10^{19}$ Nm, and 7.0, respectively, all of which were the largest among the 1996–1998, 2002–2004, 2009–2011, and 2018–2019 L-SSEs.

## Introduction

**Figure 1:**
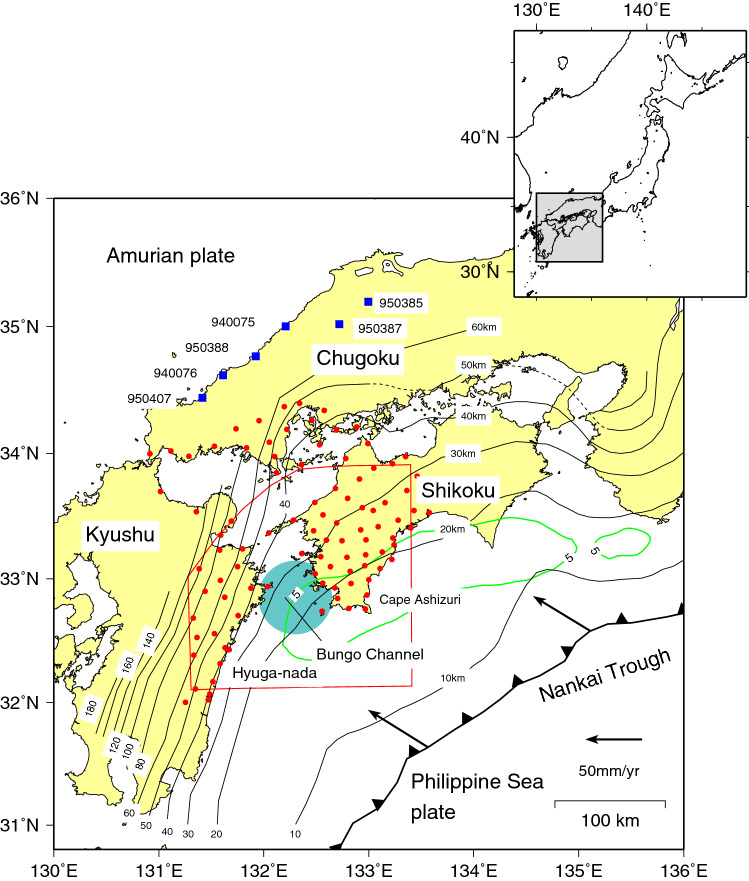
Tectonic map in and around the Bungo Channel, southwest Japan. The thin black lines represent isodepth contours of the upper surface of the Philippine Sea (PHS) plate subducting from the Nankai Trough, represented by the thick barbed black line. The 10 km, 20–50 km, and > 60 km contour lines are taken from Baba et al.^11^, Hirose et al.^12^, and Nakajima and Hasegawa^13^, respectively. The arrows indicate the plate motion velocity vector of the PHS plate with respect to the Amurian (AM) plate estimated by DeMets et al.^1^. The light-blue solid circle denotes the approximate location where the Bungo Channel slow slip events took place. The red solid circles and blue solid squares denote the GNSS stations used for the inversion analysis in this study and reference stations to calculate the common-mode error, respectively. The green line denotes the contour of 5 cm/yr of slip deficit rate of Model 1 of Yoshioka and Matsuoka^14^. The grey squared area in the inset is the study region in the map of the Japanese islands. The map was created by using the Generic Mapping Tools (GMT)^15^ (version: GMT 4.5.7, URL link: https://www.generic-mapping-tools.org/download/).

In the Bungo Channel, located between Shikoku and Kyushu in southwestern Japan, the Philippine Sea (PHS) plate is subducting beneath the Amurian (AM) plate in the northwest direction at a convergence rate of approximately 58.4 ± 1.2 mm/year^1^ (Fig. 1). Along the plate interface beneath this region, aseismic interplate slips, which are so-called long-term slow slip events (hereafter referred to as L-SSEs), have repeatedly occurred with durations of several months to a couple of years. The GNSS continuous observation system (GEONET: GNSS Earth Observation Network System) launched by the Geospatial Information Authority of Japan (GSI) in 1996 has enabled high-precision geodetic observations with high temporal resolution and the detection of such aseismic slow slip events. GNSS observation stations have been set up at approximately 1300 locations throughout the Japanese islands and play an important role in monitoring crustal deformation caused by earthquakes and volcanic activities. Previous studies have analysed the slip distributions of L-SSEs that occurred beneath the Bungo Channel in 1996–1998^2–8^, 2002–2004^4,6–9^, and 2009–2011^7,8^. Kobayashi and Yamamoto^10^ showed that L-SSEs also occurred in 1980, 1985–1986, and 1991 beneath the Bungo Channel based on levelling survey and tide gauge station data and that the L-SSEs have been repeated every 5–6 years since 1980.

Yoshioka et al.^7^ performed inversion analysis of the Bungo Channel L-SSEs that occurred in 1996–1998, 2002–2004, and 2009–2011 and compared their spatiotemporal slip distributions. In the 1996–1998 events, slip was estimated to occur beneath Cape Ashizuri on the east side of the Bungo Channel. The slip area then shifted to the central part of the Bungo Channel. The L-SSE during the period of 2002–2004 consisted of two main subevents. The first subevent occurred on the southwest side of the Bungo Channel. The second subevent can be divided into three periods. The first slip occurred in the southwest to central part of the Bungo Channel. The second slip started at the northeast side of the Bungo Channel and expanded to the southwest with the acceleration of the slip. The third slip was inferred to have been on the southwest side of the Bungo Channel. In the 2009–2011 event, the slip was estimated to start in the central part of the Bungo Channel and then shift to the southwest of the Bungo Channel. Ozawa et al.^16^ analysed the spatiotemporal slip distribution of the Bungo Channel L-SSE that occurred from 2018 to 2019. They found that slip took place at the plate boundary north of Hyuga-nada first and then occurred at the Bungo Channel.

The Bungo Channel L-SSEs have taken place downdip of the seismogenic zone adjacent to the Nankai megathrust earthquake (Fig. 1), which has been estimated to occur with a probability of 70–80% within the next 30 years^17^. Monitoring the spatiotemporal change in the behaviour of such slow earthquakes is considered to play an important role in detecting the premonitory signals of such a megathrust earthquake. From this viewpoint, it is very important to elucidate the spatiotemporal slip distribution of the 2018–2019 Bungo Chanel L-SSE and compare it with that of the past Bungo Chanel L-SSEs. In this study, we analysed the Bungo Channel L-SSE that occurred from 2018 to 2019. We used almost the same GEONET observation stations, time series data analysis method, plate geometry model, model parameterization for slip, and inversion method as in Yoshioka et al.^7^. Therefore, we can directly compare the obtained spatiotemporal slip distribution with that of the past three Bungo Channel L-SSEs and can elucidate the similarities and differences between them. In addition, Ozawa et al.^16^ showed the results of inverted spatiotemporal slip distributions of the 2018–2019 Bungo Chanel L-SSE every two months, whereas we showed them every 0.1 years. By increasing the time resolution of the presented inversion results, it is possible to clarify the spatiotemporal change in the slip in more detail.

## Data processing of GNSS time series

In this study, we used the daily coordinate positioning values (F3 solutions) of GEONET stations provided by the Geospatial Information Authority of Japan. The data period used for analysis covered 1 January 2016 to 30 June 2020. The number of observation stations used was 96 (Fig. 1), with three components at each station. Six stations (940,075, 940,076, 950,385, 950,387, 950,388, and 950,407) in the northern Chugoku district (Fig. 1), which were not affected by the L-SSE, were used as reference stations to calculate the common-mode error. We summarized the analysis procedure as follows, according to Yoshioka et al.^7^. For each day $d$ and station $S$, we calculated the residual,

1 $$\begin{array}{*{20}c} {\varepsilon_{i}^{S} \left( d \right) = P_{i}^{S} \left( d \right) - C_{i}^{S} \left( d \right)} \\ \end{array}$$

where $\varepsilon_{i}^{S} \left( d \right)$ is the residual, $P_{i}^{S} \left( d \right)$ is the time series data of the reference station after removing the coseismic steps and the steps caused by antenna exchange,${ }C_{i}^{S} \left( d \right)$ is the linear trend of the time series data of the reference station, ${\text{and}}\;i ( = 1,{ }2,\;{\text{and}}\;3$) denotes the east–west, north–south, and up-down components, respectively. The coseismic steps and the steps caused by the antenna exchange were corrected by taking the difference of the average of the previous and next 10 days. Next, the residuals of the six stations were averaged to obtain the common-mode error:

2 $$\begin{array}{*{20}c} {\hat{\varepsilon }_{i} \left( d \right) = \frac{1}{6}\mathop \sum \limits_{S = 1}^{6} \varepsilon_{i}^{S} \left( d \right)} \\ \end{array} .$$

Assuming that this error is common to all the stations used in the analysis, we removed the common-mode error from the time series data of each station.

3 $$\begin{array}{*{20}c} {\hat{O}_{i}^{T} \left( d \right) = O_{i}^{T} \left( d \right) - \hat{\varepsilon }_{i} \left( d \right)} \\ \end{array}$$

where $O_{i}^{T} \left( d \right)$ denotes the original time series data of each observation station. After removing the common-mode error, the coseismic steps and the steps caused by antenna exchange, we removed crustal movement due to plate motion, including interplate locking effects and annual and semiannual variations, assuming the following equation:

4 $$\begin{array}{*{20}c} {y\left( t \right) = a + bt + csin\left( {\frac{2\pi t}{T}} \right) + d\cos \left( {\frac{2\pi t}{T}} \right) + e\sin \left( {\frac{4\pi t}{T}} \right) + f\cos \left( {\frac{4\pi t}{T}} \right)} \\ \end{array}$$

where $T = 1$ year; the first and second terms on the right-hand side represent crustal deformation due to plate motions, including interplate coupling, assuming a linear trend; the third and fourth terms represent annual variations, and the fifth and sixth terms represent semiannual variations. $a\;{\text{to}}\;f$ are unknown parameters, and their optimal values were determined by the least-squares method. The period used for the calculation of Eq. (4) was from 1 January 2016 to 31 December 2017, when no L-SSEs took place, and these components were assumed to be invariant during the entire analysis period.

The Mw 7.0 Kumamoto earthquake occurred in central Kyushu on 16 April 2016 during this period, and postseismic deformations remained at some GNSS stations located in and around central Kyushu. We checked the corrected time series data carefully and removed such time series at some stations, which are not shown in Fig. 1, in our analysis. Then, we obtained the optimal continuous curves for three components at all GNSS stations to explain the obtained corrected daily time series data by using the superposition of cubic B-spline functions based on Akaike’s Bayesian information criterion (ABIC) minimization principle^18^. This process also enabled us to estimate the standard deviation of each component data used for the inversion of the spatiotemporal slip distribution of the 2018–2019 L-SSE. The corrected time series data show that the variance of the data due to noise in the vertical displacements is larger than that in the horizontal displacements. In this study, we divided the optimal curve into every 0.1-year equal time span and obtained the difference in displacement during the time span, which is regarded as displacement for every 0.1-year time window.

## Model

In this study, we constructed our model, following Yoshioka et al.^7^. Since the L-SSE is a slip phenomenon occurring on the plate boundary, the source region is placed on the three-dimensional upper surface of the PHS plate, as shown in Hirose et al.^12^ (Fig. 1). The size of the source region at the plate interface was 200 km × 200 km in the dip and strike directions, respectively, and 11 × 11 bicubic B-spline functions were distributed as the basis functions. The number of first-order B-spline functions in the time direction was 21 between 2018.0 and 2020.0. (Here, the number after the decimal point is not a month but a year, where 0.1 year is equal to 36.5 days.) Therefore, the total number of model parameters representing the spatiotemporal slip distribution was 5082, where the slip was represented by two components, the dip and strike directions. Here, exactly the same model setting as Yoshioka et al.^7^ was used except for the number of B-spline functions in the time direction. The standard deviations used as weights of the EW, NS, and UD components during inversion were 0.14 cm, 0.14 cm, and 0.52 cm, respectively, which were obtained using the continuous optimal curve for each component at all GNSS stations and daily corrected positioning time series data, as described in the previous section.

## Results

### Horizontal and vertical displacements associated with the 2018–2019 L-SSE

The total horizontal and vertical displacements associated with the 2018–2019 L-SSE at each station during the analysis period are shown in Fig. S2a,b, respectively. A maximum horizontal displacement up to 4.7 cm in the southeast and east-southeast directions were identified around the Bungo Channel. These are almost the opposite direction of the plate motion of the PHS plate with respect to the AM plate (Figs. 1 and S2a). Maximum subsidence and uplift of approximately 1.7 cm and 5.5 cm were identified in eastern Kyushu and southwestern Shikoku, respectively (Fig. S2b). These features indicate that thrust-type slow slip took place on the plate interface beneath the Bungo Channel.

Figure S3 shows the three-component time series data after removing the common-mode error, coseismic steps, steps caused by antenna exchange, linear trend, and annual and semiannual components at some stations in Fig. S2a. At the stations in eastern Kyushu, southeastward displacements and slight subsidence associated with the 2018–2019 Bungo Channel L-SSE began to be observed from the middle of 2018 and ended in the middle of 2019. The southeastward displacements and uplift also began to be identified at stations in southwestern Shikoku during the same period.

**Figure 2:**
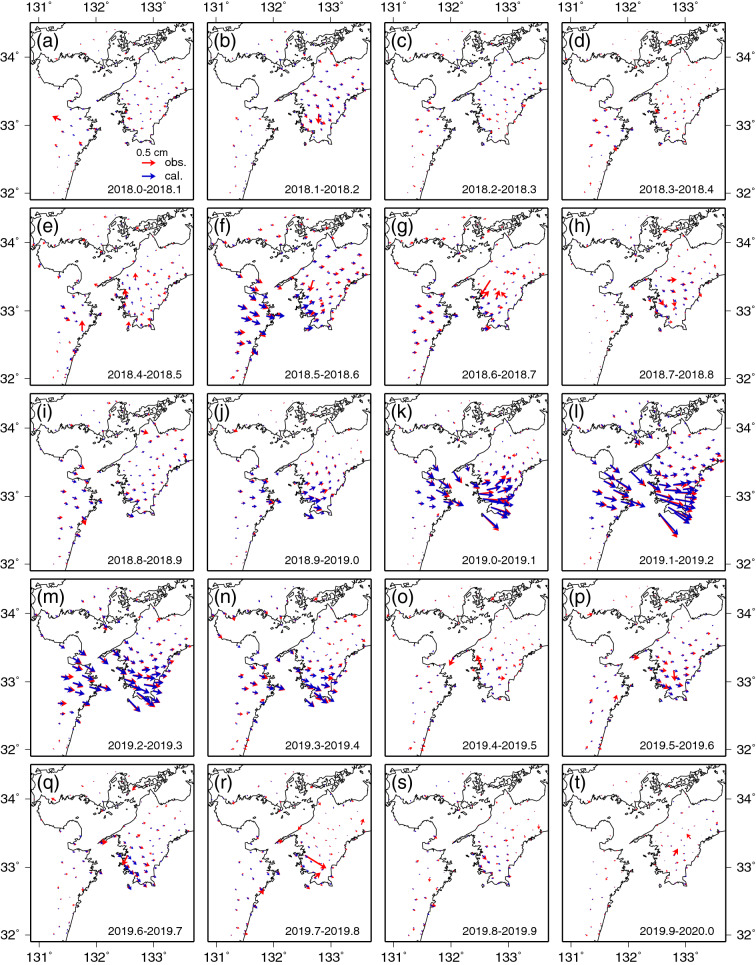
Spatial distribution of horizontal displacement fields associated with the Bungo Channel L-SSE at each 0.1-year time step between 2018.0 and 2020.0. The red and blue arrows indicate the observed displacements and the calculated displacements obtained from the spatiotemporal slip distributions shown in Fig. 4, respectively. Each 0.1-year time period is shown in each panel. The scale of 0.5 cm for the arrow is shown in (**a**). The map was created by using the Generic Mapping Tools (GMT)^15^ (version: GMT 4.5.7, URL link: https://www.generic-mapping-tools.org/download/).

The horizontal displacements at the 0.1-year time steps are shown in Fig. 2. It should be noted that all the following horizontal and vertical displacements are those during the 0.1 year concerned. There appear to be two active periods in horizontal displacements. One is the period from 2018.4 to 2018.7 (Fig. 2e–g). Displacements in the southeast to east-southeast directions are dominant at stations both in eastern Kyushu and southwestern Shikoku, especially in eastern Kyushu (Fig. 2f). The other is the period from 2018.8 to 2019.7 (Fig. 2i–q). Displacements in the same directions are dominant at stations both in eastern Kyushu and southwestern Shikoku, reaching the most activated stage in Fig. 2l. More specifically, displacements in the southeast to the east-southeast direction were observed in the northern part of eastern Kyushu, and the displacements in the southwestern part of Shikoku were larger than the first period. After southeast to east-southeast displacements were observed in both Kyushu and Shikoku between 2018.8 and 2019.4 (Fig. 2i–n), east-southeastward displacements appeared to be identified mainly in southwestern Shikoku during the subsequent period (Fig. 2p,q).

**Figure 3:**
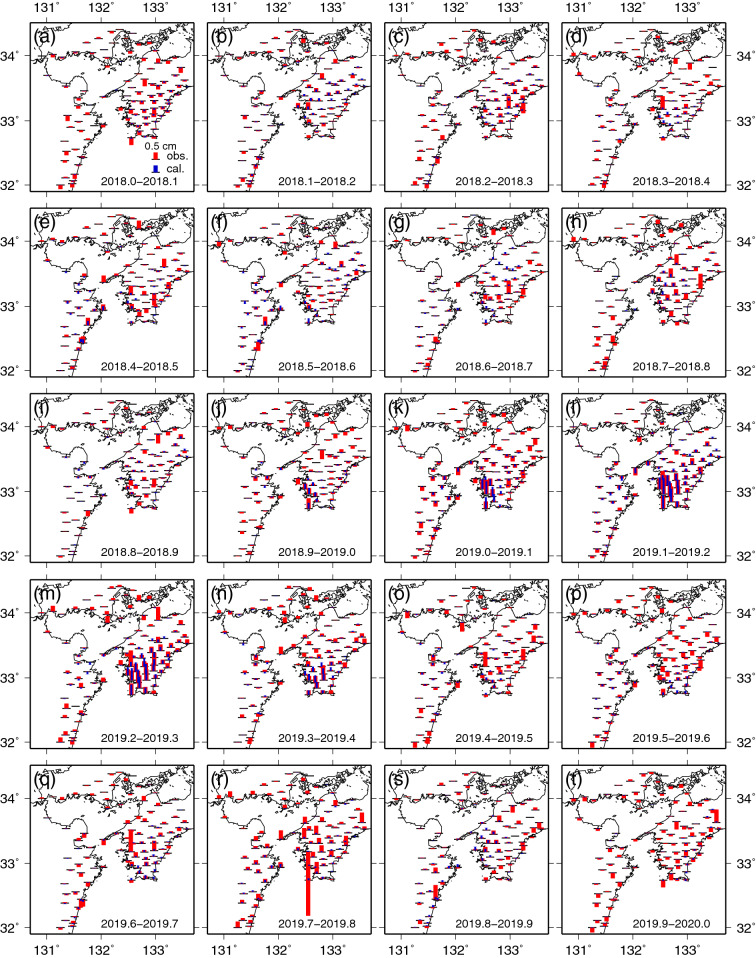
Same as Fig. 2 except for vertical displacement fields. The red and blue bars indicate the observed and calculated displacements obtained from the spatiotemporal slip distributions shown in Fig. 4, respectively. If the bars are above and below the black horizontal bars, which represent locations of the GNSS stations, they indicate uplift and subsidence, respectively. The scale of 0.5 cm for the bar is shown in (**a**). The map was created by using the Generic Mapping Tools (GMT)^15^ (version: GMT 4.5.7, URL link: https://www.generic-mapping-tools.org/download/).

The vertical displacements at the 0.1-year time steps are shown in Fig. 3. We identified that subsidence turned into slight uplift along the east coast of Kyushu between 2018.4 and 2018.7 (Fig. 3e–g). Uplift began gradually in the southwestern part of Shikoku from approximately 2018.9 (Fig. 3j) and became the largest between 2019.1 and 2019.3 (Fig. 3l,m). After that, the displacements decreased. Subsidence can be identified at most of the stations in Kyushu during almost the same period when uplift emerged in southwestern Shikoku.

From these spatiotemporal patterns, the existence of two subevents can be inferred: The first subevent took place beneath the eastern part of Kyushu (Fig. 2f), and the second subevent took place beneath the central part of the Bungo Channel (Figs. 2j–q, 3j–q).

### Spatiotemporal slip distributions associated with the L-SSE inverted from the GNSS data

**Figure 4:**
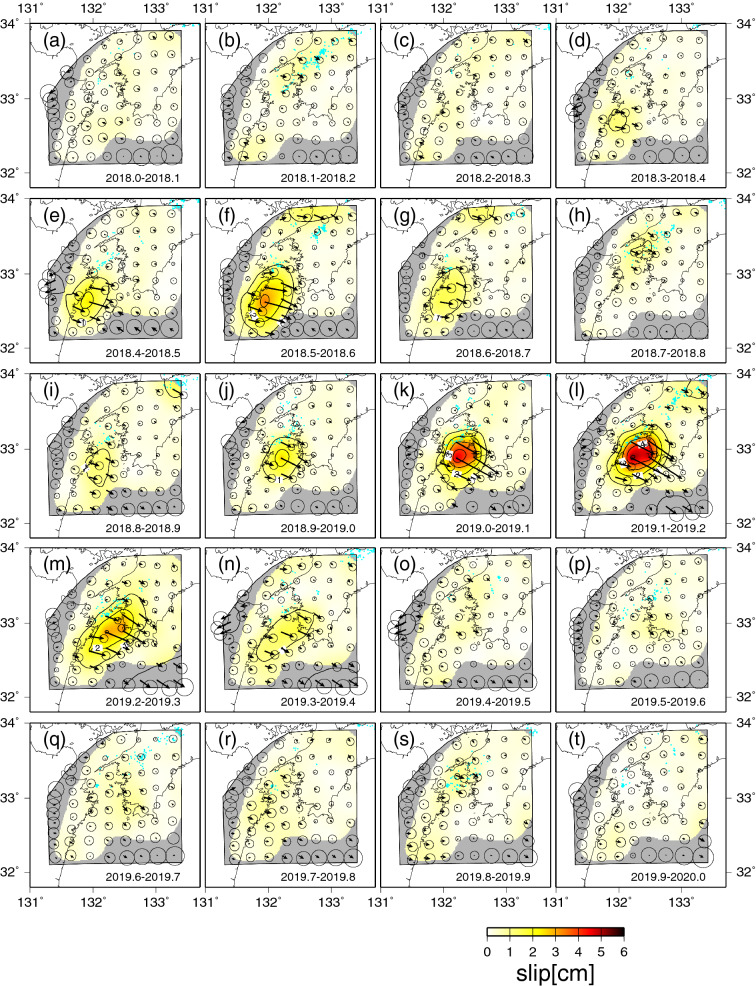
Slip distributions of the Bungo Channel L-SSE for the 0.1-year time step between 2018.0 and 2020.0. The arrows indicate the directions and amounts of the slip, and the circles at the tips of the arrows indicate the estimation errors of 1σ. The contour lines with yellowish colour show the amounts of slip with an interval of 1 cm. Areas with a resolution of less than 0.15 are masked in grey. The light blue dots indicate the epicentres of tectonic tremors that occurred during each period. We used hypocenter catalogue of tectonic tremors provided by National Research Institute for Earth Science and Disaster Resilience. Each 0.1-year time period is shown in each panel. The map was created by using the Generic Mapping Tools (GMT)^15^ (version: GMT 4.5.7, URL link: https://www.generic-mapping-tools.org/download/).

**Figure 5:**
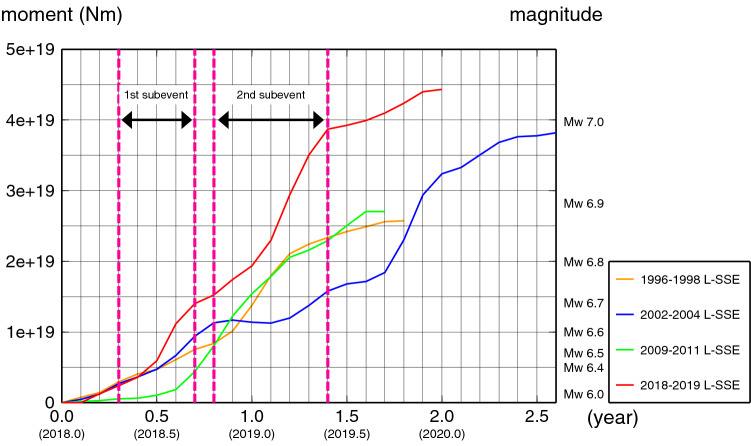
Time variation of the released seismic moment and equivalent moment magnitude for the Bungo Channel L-SSEs. The orange, blue, green, and red lines represent the 1996–1998 L-SSE, 2002–2004 L-SSE, 2009–2011 L-SSE, and 2018–2019 L-SSE, respectively. The pink dashed lines represent the durations of the two subevents of the 2018–2019 L-SSE.

The inversion analysis of the spatiotemporal slip distribution on the 3D shaped plate boundary was performed from 2018.0 to 2020.0 using the corrected GNSS time series data of each observation station. The spatiotemporal slip distributions inverted from the horizontal displacement (Fig. 2) and vertical displacement (Fig. 3) data at 0.1-year time steps are shown in Fig. 4. The maximum amount of slip, released seismic moment, and equivalent moment magnitude of the 2018–2019 L-SSE were estimated to be approximately 28 cm, $4.4 \times 10^{19}$ Nm, and 7.0, respectively (Fig. 5). The rigidity used in the calculation of seismic moments was 30 GPa.

The estimated L-SSE consists of two subevents, as expected. The first subevent was identified on the southwest side of the Bungo Channel between 2018.3 and 2018.7 (Fig. 4d–g). The maximum amount of slip, released seismic moment, and equivalent moment magnitude were estimated to be approximately 9.0 cm, $1.2 \times 10^{19}$ Nm, and 6.6, respectively (Table 1). We also estimated the maximum slip velocity by dividing the maximum slip of approximately 3.5 cm by a time window of 0.1 year, which was 35 cm/year between 2018.5 and 2018.6 (Fig. 4f).

**Table 1:** Slip-related values for the 1996–1998, 2002–2004, 2009–2011, and 2018–2019 Bungo Channel L-SSEs estimated from inversion analyses.

L-SSE	1996–1998 L-SSE	2002–2004 L-SSE		2009–2011 L-SSE	2018–2019 L-SSE	
		1st subevent	2nd subevent		1st subevent	2nd subevent
Duration	1.3 yr^(7)^	0.7 yr^(7)^	1.2 yr^(7)^	1.2 yr^(7)^	0.4 yr	0.6 yr
Maximum slip amount	15 cm^(7)^	7.3 cm^(7)^	16 cm^(7)^	19 cm^(7)^	9.0 cm	19 cm
Maximum slip velocity	28 cm/yr^(7)^	13 cm/yr^(7)^	44 cm/yr^(7)^	39 cm/yr^(7)^	35 cm/yr	54 cm/yr
Total amount of released seismic moment	$2.1 \times 10^{19}$ Nm^(7)^	$8.3 \times 10^{18}$ Nm^(7)^	$2.1 \times 10^{19}$ Nm^(7)^	$2.2 \times 10^{19}$ Nm^(7)^	$1.2 \times 10^{19}$ Nm	$2.4 \times 10^{19}$ Nm
Equivalent moment magnitude	6.8^(7)^	6.5^(7)^	6.8^(7)^	6.9^(7)^	6.6	6.8

Yoshioka et al.^7^.

The second subevent was identified beneath the central Bungo Channel area between 2018.8 and 2019.4 (Fig. 4i–n). The maximum amount of slip, released seismic moment, and equivalent moment magnitude were estimated to be approximately 19 cm, $2.4 \times 10^{19}$ Nm, and 6.8, respectively (Table 1). The maximum slip velocity was approximately 54 cm/year between 2019.1 and 2019.2 (Fig. 4l). In this subevent, the slip area expanded in the northeast-southwest direction between 2019.2 and 2019.3 (Fig. 4m). In addition, tectonic tremors appear to have been activated on the downdip side of the L-SSE occurrence region when large slips occurred beneath the Bungo Channel. After the end of the second subevent, a slight slip appears to take place between 2019.4 and 2019.8 (Figs. 4o–r, 5).

Here, we compare the displacements calculated from the slip distributions shown in Fig. 4 with the observed displacements. In Fig. 2, we can find that the calculated horizontal displacements explain the observed displacements very well both in magnitude and direction at most stations. Between 2019.5 and 2019.7, the slight displacements in the east-southeast to southeast directions identified in southwestern Shikoku were captured well by the calculations.

In Fig. 3, we show a comparison between the observed and calculated vertical displacement fields. Agreement in these data is not as good as that of the horizontal displacement fields because the data weight for the latter is larger than that for the former. There was a large difference between the observed and calculated displacements at the stations in Kyushu, but the difference was relatively small at the stations in Shikoku. In particular, the observed and calculated values were almost identical between 2019.1 and 2019.3 (Fig. 3l,m), when the southwestern part of Shikoku was greatly uplifted.

A similar tendency can also be identified from the time series data at some stations (Fig. S3). The fitting of the calculation to the corrected horizontal time series data is better than that of the vertical data, and the discrepancy tends to be slightly larger in the vertical component at the stations in Kyushu.

## Discussion

### Comparison with spatiotemporal slip distributions of the past L-SSEs beneath the Bungo Channel

L-SSEs occurred beneath the Bungo Channel in the past. Here, based on the results obtained by Yoshioka et al.^7^, we compared the characteristics of slips of the recent L-SSEs that occurred in 1996–1998, 2002–2004, and 2009–2011 with that of the L-SSE estimated in this study, all of which were identified using GNSS time series data (Table 1). The time variations of the cumulative released seismic moment of the four L-SSEs are shown in Fig. 5.

**Figure 6:**
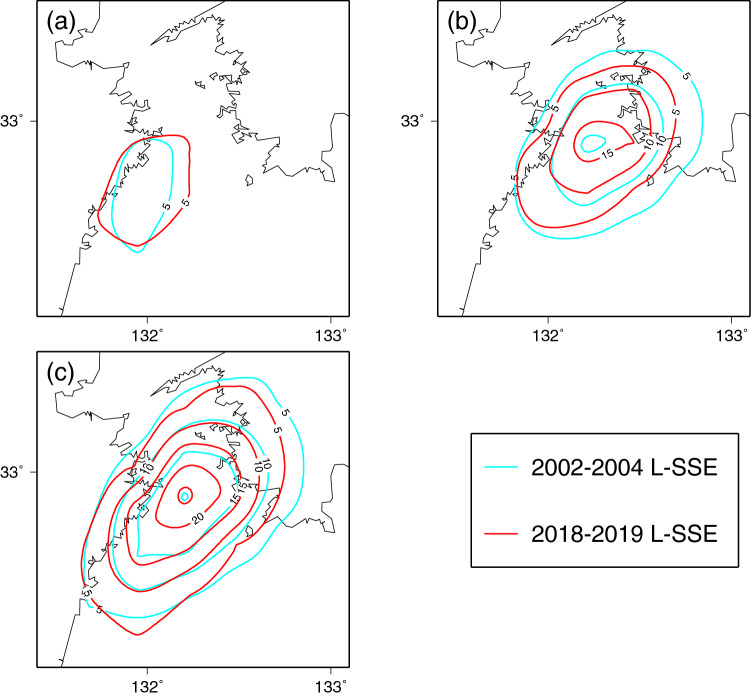
Comparison of slip distributions between the 2018–2019 Bungo Channel L-SSE (in red) obtained in this study and the 2002–2004 L-SSE (in light blue) estimated by Yoshioka et al.^7^. The contour interval is 5 cm. (**a**) Slip distributions of the two first subevents. (**b**) Slip distributions of the two second subevents. (**c**) Total slip distributions of the two L-SSEs.

The durations of the first and second subevents of the 2018–2019 L-SSE were approximately 0.4 and 0.6 years, respectively (Fig. 5, Table 1). The cumulative released seismic moment, the total slip amount, and the maximum slip velocity of the L-SSE estimated in this study were the largest among the four L-SSEs (Table 1). The recurrence interval of the L-SSEs in the past was approximately 6 years, but the estimated L-SSE in this study was approximately 8 years since the occurrence of the last L-SSE in 2009–2011. This may have affected the total slip amount. As in the case of the 2018–2019 L-SSE, there were two subevents for the 2002–2004 L-SSE. As described before, the first subevent of the L-SSE estimated in this study occurred on the southwest side of the Bungo Channel, and the second subevent took place beneath the central part of the Bungo Channel. Interestingly, the location, spatial slip distribution, and relative order in time and space in 2018–2019 were all similar to those of the 2002–2004 L-SSE (Fig. 6).

On the other hand, we found the differences between them: the maximum slip amount, the released seismic moment, and the equivalent moment magnitude of the first and second subevents of the L-SSE estimated in this study were slightly larger than those of the first and second subevents of the 2002–2004 L-SSE (Table 1, Figs. 5, 6). The time interval between the first and second subevents was 0.3 years in the 2002–2004 L-SSE, while it was 0.1 years in the 2018–2019 L-SSE, and the slip area expanded in the northeast-southwest direction in the latter half of the second subevent (Fig. 4m,n) in the 2018–2019 L-SSE. The total duration time of the two subevents was 1.0 years for the 2018–2019 L-SSE, which was shorter than that of the 2002–2004 L-SSE.

In Fig. S4, we show the relationship between the released seismic moment per 0.1 year and the number of tectonic tremors that occurred in the central part of the Bungo Channel per 0.1 year for the 2002–2004, 2009–2011, and 2018–2019 L-SSEs. Although the data are scattered, there appears to be a positive correlation between them: the number of tectonic tremors beneath the Bungo Channel tends to increase as the released seismic moment increases. The number of tectonic tremors for the 2018–2019 L-SSE appears to be smaller than that for the 2002–2004 and 2009–2011 L-SSEs, and the slope of the positive correlation for the former appears to be smaller than that for the latter, although its mechanism is unclear.

**Figure 7:**
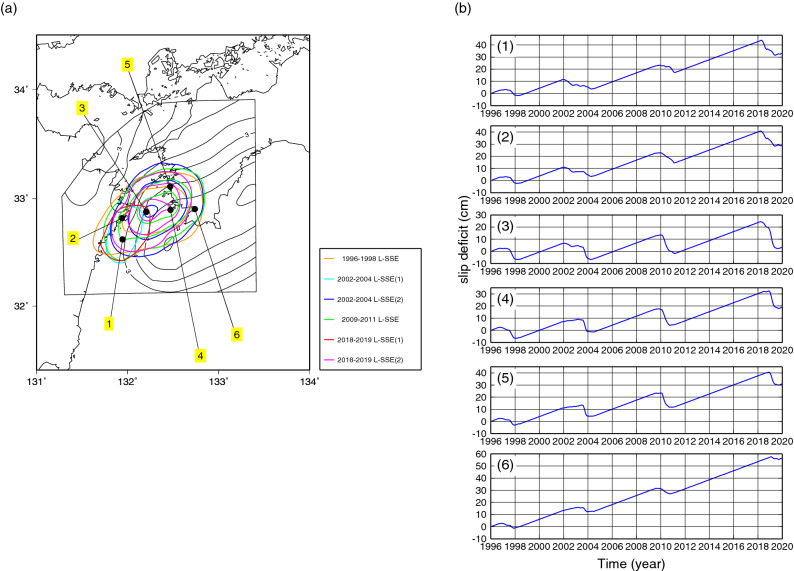
(**a**) Slip distributions for the three L-SSEs by Yoshioka et al.^7^ and the L-SSE obtained in this study and slip deficit rate distribution. The colour contour lines represent slip distributions for the 1996–1998 L-SSE (in orange) between 1996.7 and 1998.5, the 1st subevent of the 2002–2004 L-SSE (in light blue) between 2001.9 and 2004.5, the 2nd subevent of the 2002–2004 L-SSE (in blue) between 2001.9 and 2004.5, the 2009–2011 L-SSE (in green) between 2009.5 and 2011.2, the 1st subevent of the 2018–2019 L-SSE (in red) between 2018.3 and 2018.7, and the 2nd subevent of the 2018–2019 L-SSE (in pink) between 2018.8 and 2019.4. The contour interval is 5 cm. The black contour lines denote the slip deficit rate distribution of Model 1 of Yoshioka and Matsuoka^14^. Their contour interval is 1 cm/yr. The solid black numbered circles are locations to show the slip deficit budget in (**b**). (**b**) The cumulative amounts of slip deficit since 1996.5, which were calculated from the amount of slip deficit rate of Model 1 of Yoshioka and Matsuoka^14^ and the slip amounts associated with the 1996–1998, 2002–2004, 2009–2011, and 2018–2019 L-SSEs. (1)–(6) represent the slip deficit budget at the locations shown in (**a**). Positive and negative directions denote the accumulation and release of slip deficits, respectively. The map was created by using the Generic Mapping Tools (GMT)^15^ (version: GMT 4.5.7, URL link: https://www.generic-mapping-tools.org/download/).

In Fig. 7a, we show slip distributions of the past three L-SSEs by Yoshioka et al.^7^ and the slip distribution of the 2018–2019 L-SSE obtained in this study together with slip-deficit-rate distribution by Yoshioka and Matsuoka^14^. It should be noted that the slip distribution of the 2018–2019 L-SSE is almost identical to those of the past three L-SSEs. Figure 7b shows the cumulative amounts of slip deficit since 1996.5, which were calculated from the slip deficit rate of Model 1 of Yoshioka and Matsuoka^14^ and the slip amounts associated with the 1996–1998, 2002–2004, 2009–2011, and 2018–2019 L-SSEs at the six locations shown in Fig. 7a. Assuming that the slip deficit rate in this region during the period since 1996.5 has been constant and the same as that obtained by Yoshioka and Matsuoka^14^, we noticed that the accumulated slip deficit during the period since the occurrence of the 2009–2011 L-SSE was almost released at location (3) by the slip of the 2018–2019 L-SSE. Approximately half of the accumulated slip deficit was released at locations (1), (2), (4), and (5). On the other hand, little accumulated slip deficit was released at location (6). We also found that the timing of release of the accumulated slip deficit was different, depending on the locations: it started from the beginning to the middle of 2018 at locations (1)–(3), which are located in the western part of the Bungo Channel. On the other hand, the timing was from the end of 2018 to the beginning of 2019 at locations (4)–(6), which are located in the eastern part of the Bungo Channel.

Matsuzawa et al.^19^ numerically simulated Bungo Channel L-SSEs, incorporating the 3D configuration of the subducting PHS plate. They adopted a rate- and state-dependent friction law with cut-off velocities, assuming frictional parameter distribution: Effective normal stress starts to decrease in the L-SSE occurrence region at shallower depths rather than the surrounding region, and the a–b value is kept negative within the L-SSE region in the Bungo Channel (See Fig. 1d in Matsuzawa et al.^19^). As a result, their numerical model successfully reproduced the recurrence intervals of several years and locations of L-SSEs in the Bungo Channel. Their results indicate that such spatial distributions of the frictional parameter are needed to take place L-SSEs with intervals of several years as shown in this study. They also noted that as L-SSEs occur between the locked region of megathrust events and the downdip ETS region, slip behaviour in this region should be largely related to the stress state of the megathrust earthquake cycle. Further observation and comparison with numerical simulations are important for understanding the stress accumulation and nucleation process of megathrust earthquakes.

### Comparison with a previous study on spatiotemporal slip distributions of the 2018–2019 Bungo Channel L-SSE

Next, we compared the spatiotemporal slip distributions of the 2018–2019 Bungo Channel L-SSE obtained in this study with those obtained in a previous study. Ozawa et al.^16^ performed a network inversion filter by McGuire and Segall^20^ and found that the first subevent was inferred on the north side of Hyuga-nada from June 2018 to October 2018 and that the second subevent was estimated beneath the Bungo Channel from October 2018 to August 2019. The locations of the two subevents were almost the same as those obtained in this study. In Ozawa et al.^16^, the second subevent also expanded in the northeast and southwest directions, as shown in Fig. 4n. The maximum amount of slip was estimated to be approximately 30 cm by Ozawa et al.^16^, which was almost the same as that estimated in this study. The moment magnitude of the L-SSE was estimated to be 7.0, which was also consistent with the results of this study.

However, there were also some discrepancies between the two studies. The first subevent started between 1 June 2018 and 1 August 2018 in Ozawa et al.^16^, whereas it initiated between 2018.3 and 2018.4 (20 April 2018 and 26 May 2018) in this study. In addition, between 1 April 2019 and 1 June 2019, the slip near the centre of the Bungo Channel decreased, and slip split to the southwest side of Shikoku and the north side of Hyuga-nada in Ozawa et al.^16^, whereas there was no decrease in the slip near the centre of the Bungo Channel in this study (Fig. 4m,n). Furthermore, the slip decreased in the period from June 2019 to August 2019 in Ozawa et al.^16^, but in this study, a slight slip whose amount was larger than its estimation error (1σ) was identified in the period from 2019.4 to 2019.8, which also temporally coincided with tectonic tremors (Fig. 4o–r). This suggests that the slip continued even after August 2019. These differences may be caused by differences in the temporal resolution of the presented inversion results, the difference in the correction of the GNSS time series data, and the difference in the inversion method. With regard to the temporal resolution of the presented inversion results, Ozawa et al.^16^ showed the temporal change in the slip distribution every two months, while in this study, we showed them every 0.1 year. So, the slip amount at each presented time step by Ozawa et al.^16^ was expected to be larger than that of this study. For the correction of the GNSS time series data, Ozawa et al.^16^ used the estimation period of the annual variations from 2000 to 2018 and the linear trend from 1 January 2017 to 1 January 2018, while in this study, the estimation period of both the annual and semiannual variations and the linear trend was from 1 January 2016 to 31 December 2017. For the inversion method, Ozawa et al.^16^ used the constraints that the slip vectors should be within the range of 10° from the direction opposite to the motion of the PHS plate with respect to the AM plate. They also assumed that slip motion on a plate interface varies smoothly with time, and its second derivative is white noise. On the other hand, in this study, we used the three prior constraints described in the “Methods” section.

## Conclusions

In this study, we analysed the spatiotemporal slip distributions of the long-term slow slip event that occurred on the plate boundary beneath the Bungo Channel from 2018 to 2019 using GNSS time series data. We used the inversion method proposed by Yoshioka et al.^7^ with the three prior constraints. Significant results obtained in this study can be summarized as follows:

1. Regarding the 2018–2019 L-SSE analysed in this study, the maximum slip velocity of 54 cm/year, slip amount of 28 cm, total amount of released seismic moment of $4.4 \times 10^{19}$ Nm, and equivalent moment magnitude of 7.0 were all the largest among the four L-SSEs that were recorded by GNSS time series data. The slip amount may be related to the longest recurrence interval of 8 years since the occurrence of the last L-SSE.
2. The 2018–2019 L-SSE consists of two subevents. The first subevent occurred on the southwest side of the Bungo Channel, with a duration of 0.4 years, maximum slip of approximately 9.0 cm, maximum slip velocity of approximately 35 cm/year, released seismic moment of 1.2 × 10^19^ Nm, and equivalent Mw of 6.6. The second subevent took place near the centre of the Bungo Channel, with a duration of 0.6 years, maximum slip of approximately 19 cm, maximum slip velocity of approximately 54 cm/year, released seismic moment of 2.4 × 10^19^ Nm, and equivalent Mw of 6.8.
3. Tectonic tremors appear to have been activated on the downdip side of the L-SSE occurrence region when large slips occurred beneath the Bungo Channel.
4. Compared with the past L-SSEs beneath the Bungo Channel, the location, spatial slip distribution, and relative order in time and space of the two subevents are similar to those of the 2002–2004 L-SSE.

## Methods

We performed an inversion analysis for the crustal deformation associated with the 2018–2019 Bungo Channel L-SSE by the following procedure using corrected GNSS time series data. The medium is assumed to be a semi-infinite homogeneous perfect elastic body.

In this study, we used the inversion method proposed by Yoshioka et al.^7^ with three prior constraints: (1) the spatial slip distribution is smooth, (2) the slip directions are oriented in the direction of plate convergence, and (3) the temporal change in the slip is smooth. The slip distribution is represented by a superposition of bicubic B-spline functions, and the temporal evolution is represented by a superposition of first-order B-spline functions. In the following, we briefly summarize the inversion method according to Yoshioka et al.^7^. The relationship between the model parameters representing the spatiotemporal slip distribution and the observed data can be expressed by the following equation:

5 $$\begin{array}{*{20}c} {\left[ {\begin{array}{*{20}c} {\mathbf{d}} \\ 0 \\ 0 \\ 0 \\ \end{array} } \right] = \left[ {\begin{array}{*{20}c} {\mathbf{H}} \\ {\hat{\alpha }{\mathbf{A}}} \\ {\hat{\beta }{\mathbf{B}}} \\ {\hat{\gamma }{\mathbf{G}}} \\ \end{array} } \right]\left[ {\varvec{a}} \right]} \\ \end{array}$$

where **d** is the vector of observed displacement data, **H** is a matrix representing the relationship between the unit slip on the fault plane and the displacement at each GNSS observation station, ***a*** is a vector of model parameters, **A**, **B**, and **G** are matrices representing the above-described first, second and third constraints, respectively. $\hat{\alpha },\;\hat{\beta },\;{\text{and}}\;\hat{\gamma }$ are the optimal values of the hyperparameters and represent the optimal values of the weight of the constraints. These hyperparameters are determined uniquely and objectively based on the ABIC minimization principle^16^. Using covariance matrix σ^2^E originated from data errors, a covariance matrix **C** of the estimation error for the estimated model parameter vector can be obtained as follows:

6 $$\begin{array}{*{20}c} {{\mathbf{C}} = \hat{\sigma }^{2} \left( {{\mathbf{H}}^{{\text{T}}} {\mathbf{E}}^{ - 1} {\mathbf{H}} + \hat{\alpha }^{2} {\mathbf{A}}^{{\text{T}}} {\mathbf{A}} + \hat{\beta }^{2} {\mathbf{B}}^{{\text{T}}} {\mathbf{B}} + \hat{\gamma }^{2} {\mathbf{G}}^{{\text{T}}} {\mathbf{G}}} \right)^{ - 1} } \\ \end{array}$$

where $\hat{\sigma }^{2}$ is described in Yoshioka et al.^7^. A resolution matrix can be defined as

7 $$\begin{array}{*{20}c} {{\mathbf{R}} = \left( {{\mathbf{H}}^{{\text{T}}} {\mathbf{E}}^{ - 1} {\mathbf{H}} + \hat{\alpha }^{2} {\mathbf{A}}^{{\text{T}}} {\mathbf{A}} + \hat{\beta }^{2} {\mathbf{B}}^{{\text{T}}} {\mathbf{B}} + \hat{\gamma }^{2} {\mathbf{G}}^{{\text{T}}} {\mathbf{G}}} \right)^{ - 1} {\mathbf{H}}^{{\text{T}}} {\mathbf{H}}} \\ \end{array} .$$

The value of the resolution at each solved point on the plate boundary is defined by the diagonal component of the resolution matrix as

8 $$\begin{array}{*{20}c} {R = \sqrt {\frac{{\left( {R^{P} } \right)^{2} + \left( {R^{S} } \right)^{2} }}{2}} } \\ \end{array}$$

where $R^{P}$ and $R^{S}$ are the resolutions in the plate convergence direction and perpendicular to the plate convergence direction, respectively.

In this study, we evaluated the reliability of the inverted slip distributions using the calculated estimation errors and resolutions.

The optimal values of the hyperparameters representing the weights of the three prior constraints $\hat{\alpha },\;\hat{\beta },\;{\text{and}}\;\hat{\gamma }$ used in this study were obtained as $7.4 \times 10^{ - 2}$, $6.7 \times 10^{ - 1}$, and $7.4 \times 10^{ - 2}$, respectively.

## Supplementary Information

Supplementary Information.
