# Supplementary material for: Spatiotemporal slip distributions associated with the 2018–2019 Bungo Channel long-term slow slip event inverted from GNSS data
Source: Sci Rep. 2022 Jan 10;12:343. doi: 10.1038/s41598-021-03982-6 (PMC8748931; doi:10.1038/s41598-021-03982-6)
Supplement: Supplementary file 1 — Supplementary Information. [file 41598_2021_3982_MOESM1_ESM.docx]

Supplementary information for

“Spatiotemporal slip distributions associated with the 2018-2019 Bungo Channel long-term slow slip event inverted from GNSS data”

Yukinari Seshimo^1,^* and Shoichi Yoshioka^2,1^

^1^Department of Planetology, Graduate School of Science, Kobe University, Rokkodai-cho 1-1, Nada ward, Kobe 657-8501, Japan

^2^Research Center for Urban Safety and Security, Kobe University, Rokkodai-cho 1-1, Nada ward, Kobe 657-8501, Japan

*corresponding author

TEL: +81-80-2624-6982

FAX: +81-78-803-6598

e-mail: y.seshimo@stu.kobe-u.ac.jp

Contents

　Text

Figures and figure captions S1-S4

References

**Effects of removing common-mode error**

Fig. S1 shows three-component time series data at two stations located in southwestern Shikoku and eastern Kyushu before and after removing common-mode error during the period from 1 January 2016 to 30 June 2020. By removing the common-mode error, the variability of the data has been reduced for all three components. In particular, the steps in the NS component in early 2017 before removing the common-mode error, which were also identified at most stations, disappeared after removing the common-mode error.

**Figure S1**


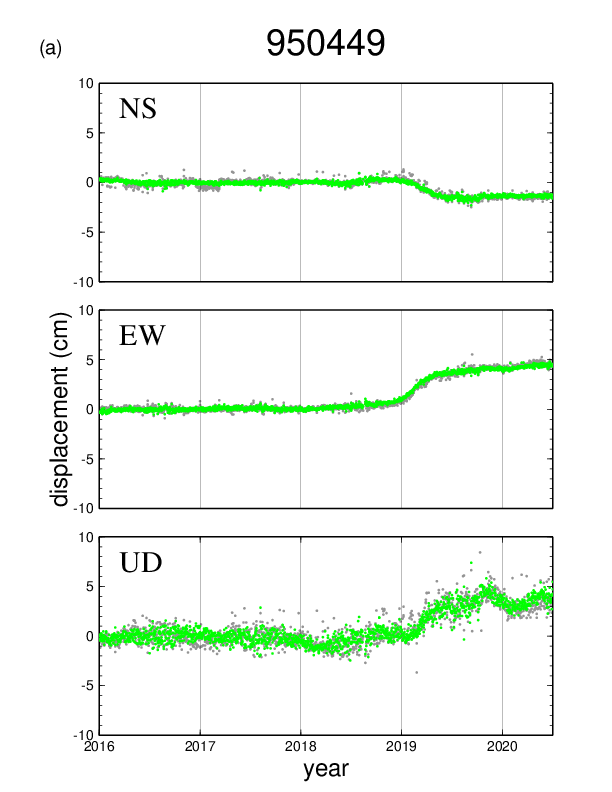


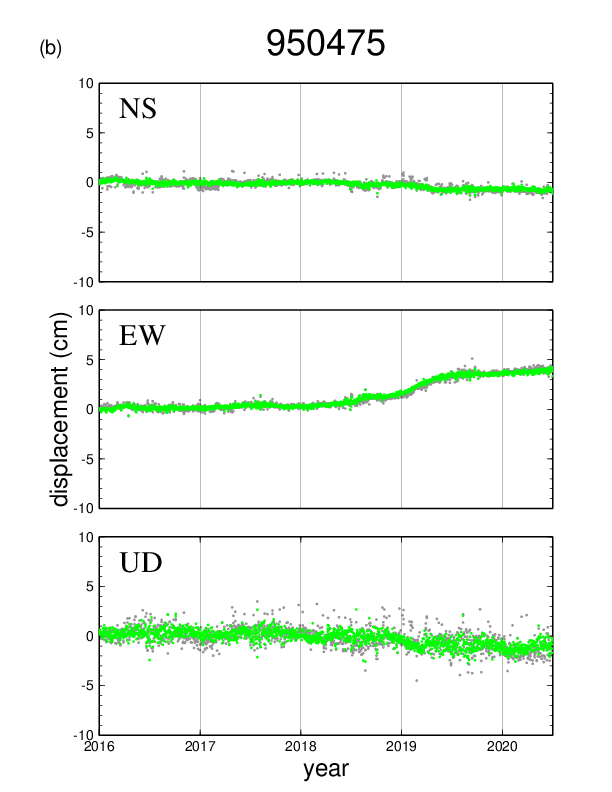


Figure S1　Time series data before (grey dots) and after (green dots) removing common-mode error during the period from 1 January 2016 to 30 June 2020. Top panel: north-south component. Positive and negative displacements denote northward and southward displacements, respectively. Middle panel: east-west component. Positive and negative displacements denote eastward and westward displacements, respectively. Bottom panel: up-down component. Positive and negative displacements denote upward and downward displacements, respectively. (a) 950449 station located in southwestern Shikoku whose exact location is shown in Figure S2. (b) 950475 station located in eastern Kyushu whose location is shown in Figure S2.


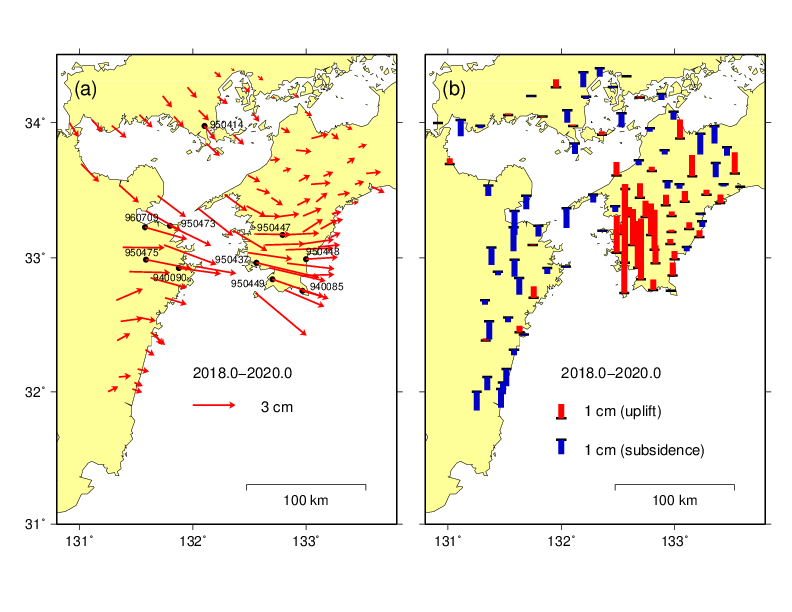
**Figure S2**

Figure S2　Total displacement fields associated with the L-SSE obtained at GNSS stations during the period from 2018.0 to 2020.0. (a) Total horizontal displacement field. The numbered stations indicate the locations of the time series data shown in Fig. S3. (b) Total vertical displacement field. The black horizontal bars indicate the locations of the GNSS stations, and the red and blue bars indicate uplift and subsidence, respectively. The map was created by using the Generic Mapping Tools (GMT)^1^ (version: GMT 4.5.7, URL link:

https://www.generic-mapping-tools.org/download/).


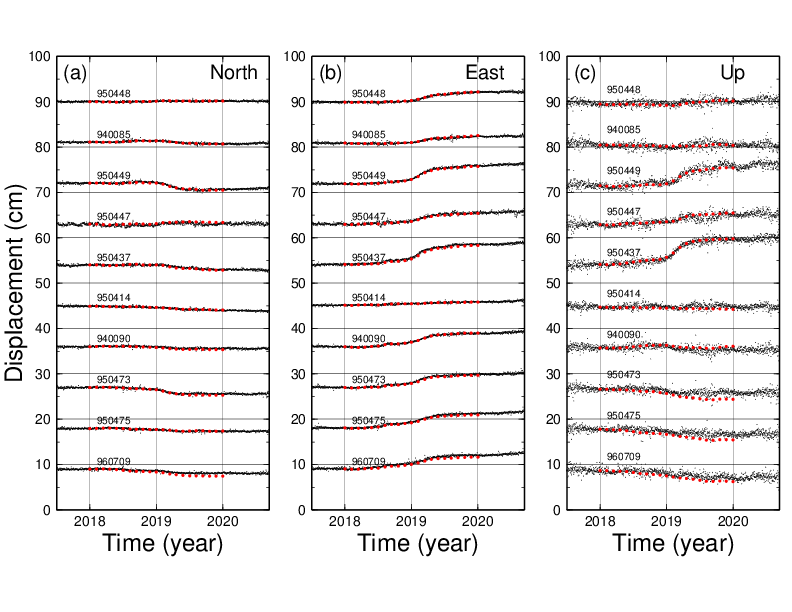
**Figure S3**

Figure S3 　GNSS time series data including displacements associated with the 2018-2019 L-SSE at the stations shown in Fig. S2(a). The black dots indicate the daily data after removing the coseismic steps, steps due to antenna exchange, linear trends, annual and semiannual variations, and the common-mode error. The red dots denote the displacement at each station every 0.1 year calculated from the inverted spatiotemporal slip distributions shown in Fig. 4. (a) North-south component. (b) East-west component. (c) Vertical component.


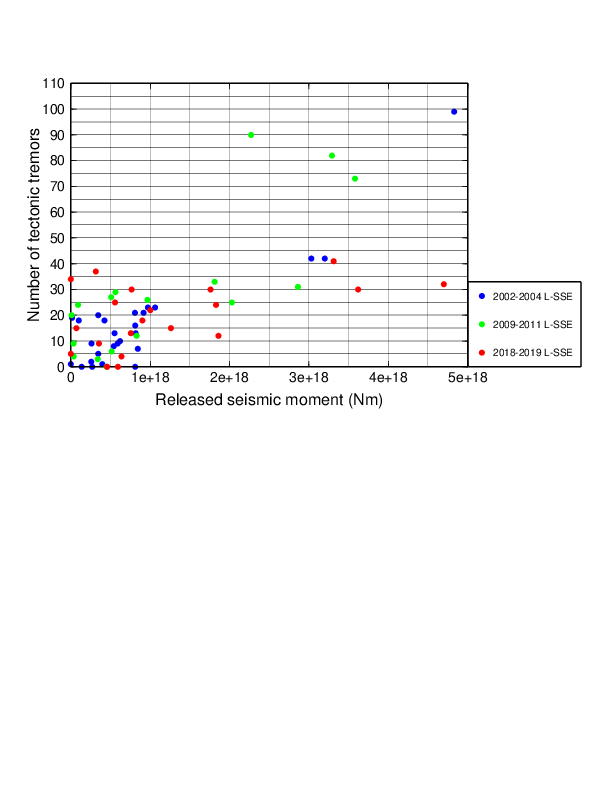
**Figure S4**

Figure S4 Relationship between released seismic moment per 0.1 year and number of tectonic tremors that occurred in the central part of the Bungo Channel per 0.1 year. The blue, green, and red circles represent the 2002–2004, 2009–2011, and 2018–2019 L-SSEs, respectively. We used a hypocentre catalogue of tectonic tremors provided by the National Research Institute for Earth Science and Disaster Resilience.

**References**

1. Wessel, P., & Smith, W.H.F. New, improved version of the generic mapping tools released. EOS Trans. AGU 79, 579 (1998).
